# Conditional associations of sex steroid hormones with C-reactive protein levels in American children and adolescents: evidence from NHANES 2015-2016

**DOI:** 10.3389/fendo.2024.1431984

**Published:** 2024-09-24

**Authors:** Zhisheng Zhu, Xingong Lin, Chaoyang Wang, Shize Zhu, Xianying Zhou

**Affiliations:** Plastic Surgery, The Second Affiliated Hospital of Fujian Medical University, Quanzhou, China

**Keywords:** children, adolescents, NHANES, sex steroid hormones, C-reactive protein

## Abstract

**Background:**

The relationship between sex steroid hormones and high-sensitivity C-reactive protein(hs-CRP) levels in American children and adolescents is understudied. This research will examine this association.

**Methods:**

The study conducted a data analysis from the National Health and Nutrition Examination Survey (NHANES) 2015-2016, adjusting multiple linear regression models with R 4.2.2 and EmpowerStats. A total of 1,768 children and adolescents were surveyed. Data collection involved measurements of serum levels of testosterone, estradiol, sex hormone-binding globulin (SHBG) and hs-CRP.

**Results:**

With the increase in testosterone, a brief rise (β=0.082, *P*=0.047) followed by an overall decline (β=-0.028, *P*=0.023) in hs-CRP was observed in the Male Prepubertal population, while a continuous decline (β=-0.002, *P*<0.05) was seen in the Male Pubertal group. A positive correlation (β=0.047, *P*<0.05) was found between testosterone and hs-CRP in the Female Prepubertal population, whereas no significant association (β=0.002, *P*>0.05) was detected in the Female Pubertal group. A significant inverse correlation was observed between estradiol and hs-CRP solely in the Female Pubertal group (β=-0.002, *P*<0.05), while no association was found in other populations. An inverse relationship between SHBG and hs-CRP was consistently noted across all groups: Male Prepubertal, Male Pubertal, Female Prepubertal, and Female Pubertal.

**Conclusions:**

The association between sex steroid hormones and high-sensitivity C-reactive protein (hs-CRP) levels among American children and adolescents is conditional and influenced by multiple factors.

## Introduction

C-reactive protein (CRP), predominantly produced by hepatocytes in the liver, is a homopentameric protein involved in the acute-phase inflammatory response, and its expression is noticeably heightened during inflammation, such as that caused by rheumatoid arthritis, specific cardiovascular disorders, and infections (1). Estrogens are generally observed to exert an anti-inflammatory influence, potentially enhancing outcomes in severe infections and during wound healing processes (2). However, the literature also indicates that the effects of estrogens on inflammation can vary, being pro-inflammatory, anti-inflammatory, or both, depending on specific cytokines, cell types, and variations in estrogen receptor expression (3). Conversely, testosterone levels are consistently found to have anti-inflammatory effects, as they inhibit adipocyte expansion, differentiation, and function, reduce the production of various cytokines, and promote adiponectin secretion (4). Furthermore, estrogen is speculated to alter CRP concentrations, with evidence indicating a notable impact of hormone replacement therapy (HRT) on CRP levels in the elderly population (1).

Prior research has revealed a complicated and varied relationship between sex steroid hormones and CRP levels in adults. In men, there is generally an inverse association between CRP and key hormonal markers like total testosterone, free testosterone, and SHBG, while the relationship with estradiol is often insignificant (5). Interestingly, estrogen treatment in certain male groups could potentially elevate CRP levels (6). In women, the relationship varies greatly with hormonal and menopausal status, presenting an intricate pattern of positive, negative, or even non-significant correlations between different sex hormones and CRP levels (7–9).

To date, only a single study targeting the adolescent population (aged 12-16 years) has examined the relationship between sex hormones and C-reactive protein. It found a negative correlation between testosterone and high-sensitivity C-reactive protein(hs-CRP) in adolescent boys, but no correlation in girls. In both sexes, a negative association was found between SHBG and hs-CRP (10).

The study of sex steroid hormones in children and adolescents has been attracting increasing scholarly attention (11, 12). Concurrently, existing findings regarding the relationship between these hormones and CRP remain contentious. It is noteworthy that there is a significant scarcity of research, particularly in the age group of 6-11 years. This oversight is notable given the critical developmental changes that occur during these years, which could potentially modulate the influence of sex hormones on inflammation differently than in adults. Understanding these relationships is crucial, as it could enable earlier and more effective interventions for diseases related to inflammation and hormonal imbalances, and transform preventative health strategies. To bridge this research gap, our study proposes to harness the 2015-2016 NHANES data. The objective is to elucidate the potential associations between sex steroid hormones and hs-CRP levels within the population of American children and adolescents. Given the cross-sectional nature of NHANES, our study focuses on identifying patterns and associations rather than causative links, which is a pertinent approach when dealing with observational datasets. Building on this framework, our hypothesis posits that the relationships between sex hormone levels and high-sensitivity C-reactive protein (hs-CRP) vary across different stages of puberty in children and adolescents. We anticipate unique patterns in the correlation between sex hormones and hs-CRP across different genders of children and adolescents before puberty. During puberty, this relationship is expected to mirror the complex interactions observed in adult studies. These findings will provide new insights into the correlations between sex hormones and inflammatory markers during different developmental stages.

## Participants and methods

### Study design and participants

**Figure 1:**
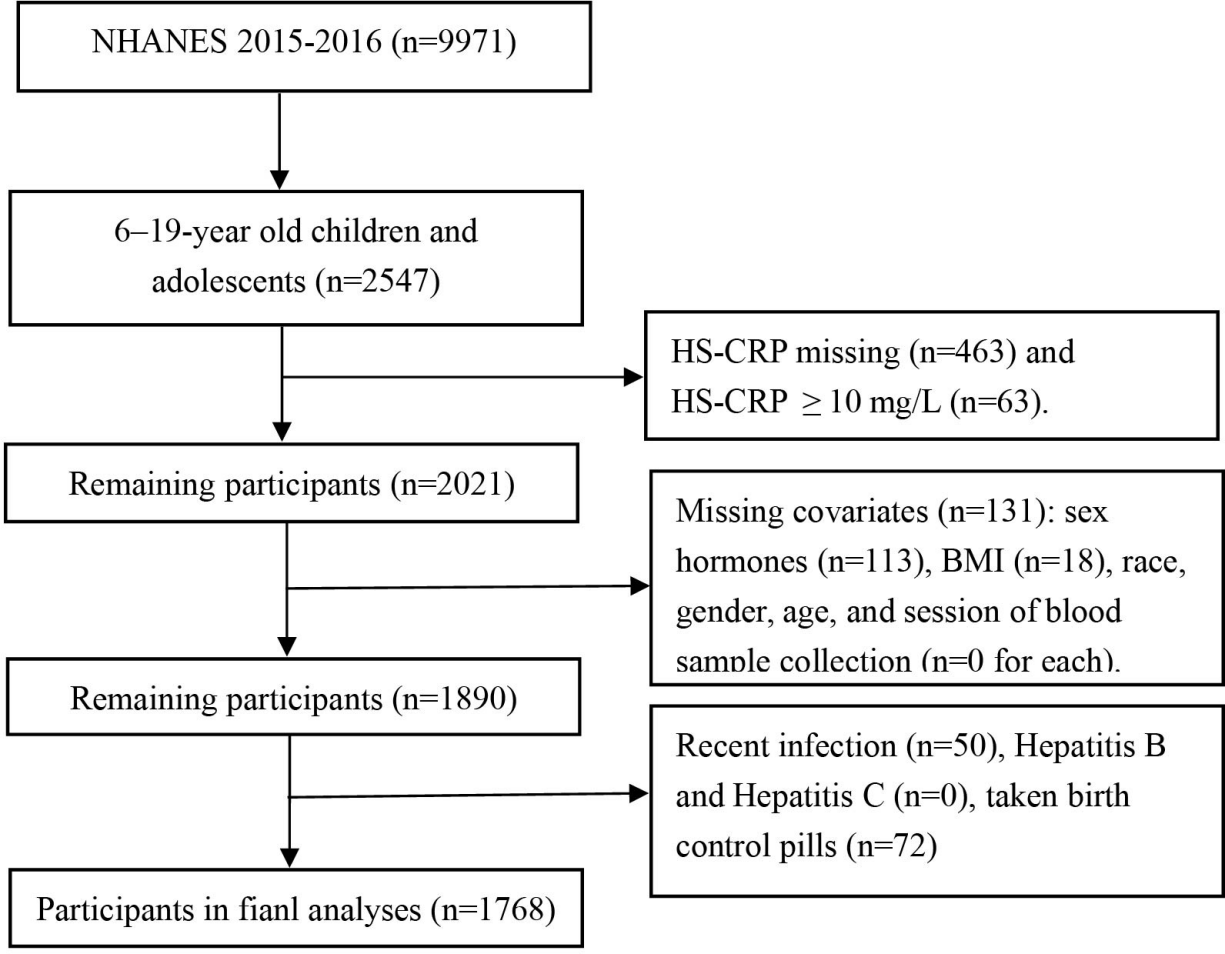
Flowchart for selecting the study population. NHANES, The National Health and Nutrition Examination Survey.

The National Health and Nutrition Examination Survey (NHANES) is a sequence of cross-sectional studies carried out in the United States, assessing the health and nutritional conditions of both adults and children. In contrast to other research, NHANES integrates physical exams and interviews for comprehensive data gathering. The ethical aspects of each part of the study were reviewed and approved by the National Center for Health Statistics Ethics Review Board, and every participant gave their written informed consent (13). For the purpose of our research, we extracted data from 1,768 children(6-11 years) and adolescents(12-19 years) who had hs-CRP, serum total testosterone (TT), estradiol (E_2_), and sex hormone binding globulin (SHBG) records in the 2015-2016 NHANES dataset. The process of our selection is depicted in **Figure 1**.

### Sex hormone indicators as measured in NHANES

The measurement of testosterone, estradiol, and SHBG in NHANES were conducted by NHANES researchers, following standardized procedures.

The acquisition of testosterone and estradiol involves four principal steps: Dissociation of the analytes from binding proteins, extraction of the analytes from the sample matrix, removal of potentially interfering compounds, and quantitation of the analytes by isotope dilution high performance liquid chromatography tandem mass spectrometry (ID-LC-MS/MS) using stable isotope labeled internal standards and external calibrators. Isolation of the analytes is achieved using liquid-liquid extraction. ID-LC-MS/MS is performed with a triple quadrupole mass spectrometer using electrospray ionization in positive ion mode for testosterone, and negative ion mode for estradiol. Estradiol and testosterone are identified based on chromatographic retention time and on specific mass to charge ratio transitions using selected reaction monitoring (SRM). A ^13^C isotope-labeled testosterone and a ^13^C isotope-labeled estradiol are used as internal standards.

The method for measuring Sex Hormone Binding Globulin (SHBG) is based on the reaction of SHBG with immuno-antibodies and chemo-luminescence measurements of the reaction products. It consists of 2 incubation steps and a chemiluminescent measurement via photomultiplier tube that spans 18 minutes.The first incubation period begins by sandwiching the sample of SHBG containing serum between a biotinylated monoclonal SHBG-specific antibody and a monoclonal SHBG-specific antibody that is labeled with ruthenium. The second incubation entails the addition of streptavidin-coated microparticles to the sample mixture. The microparticles bind to the solid phase via biotin and streptavidin interactions. The resulting sample mixture is then aspirated into a measuring cell that is subjected to a magnetic field. This captures the microparticles on an electrode. The remains of the sample mixture are subsequently washed out of the measuring cell. A voltage is applied to the electrode causing a chemiluminescent reaction that is measured by a photomultiplier tube. The readings are compared to an instrument- and lot-specific calibration curve.

### High-sensitivity C-reactive protein as measured in NHANES

High-sensitivity C-reactive protein (hs-CRP) levels were obtained from the NHANES 2015-2016 dataset, where the hs-CRP measurements were carried out by NHANES staff using a dual-agent immunoturbidimetric system at designated laboratories. Initially, a sample is amalgamated with a Tris buffer followed by an incubation period. Then, the second reagent, comprising latex particles that are coated with mouse anti-human CRP antibodies, is introduced. Upon exposure to circulating CRP, the latex particles tend to clump together, leading to the formation of immune complexes. These formed complexes induce an increase in the scattering of light, which is directly proportional to the CRP concentration. The resulting light absorption from the scattered light is then measured against a pre-established CRP standard curve to determine the concentration of CRP (14).

### Covariates

The selection of covariates in this study was based on previous research on sex hormones (5, 8, 9), and included age (continuous), race (Mexican American, other Hispanic, Non-Hispanic white, Non-Hispanic black, Non-Hispanic Asian, or other race), education level (6th and below 6th grade, above 6th grade), poverty income ratio (PIR, continuous), body mass index (BMI, continuous), diabetes (categorical), session of blood sample collection (morning, afternoon, or evening), total cholesterol(TC, continuous), and physical activity(non-activity, 0.1-0.9 hour/week, 0.1-0.9 hour/week, 1.0-3.4 hour/week, 3.5-5.9 hour/week, and ≥6 hour/week). To identify participants with diabetes, any of the following characteristics were used: (a) hemoglobin A1C concentration ≥ 6.5% or a fasting plasma glucose level ≥126 mg/dL; (b) for those who answered “yes” to the following questions: ‘Take diabetic pills to lower blood sugar?’ or ‘Doctor told you have diabetes?’ or ‘Taking insulin now?’ (15).

### Adjustment for pubertal status

It is noteworthy that segmenting the participants aged 6-19 years into categories of children and adolescents based purely on age could result in blending prepubescent and pubescent individuals within each group. Consequently, this could give rise to unusually high or low concentrations of sex hormones within each category, which could skew the relationships between sex hormones and hs-CRP during regression analysis. In addition, the influence of sex hormones on hs-CRP may vary with puberty status. In an effort to mitigate this concern, we subdivided the participants into two categories, namely pubertal and prepubertal groups, based on their serum sex hormone levels and menarcheal status. Participants who displayed levels of TT ≥ 50 ng/dL (in the case of males) or E_2_ ≥ 20 pg/mL (in the case of females), or had initiated menstrual cycles (applicable to females), were classified under the ‘pubertal group’. The remaining participants, who did not meet these criteria, were assigned to the ‘prepubertal group’ (16).

### Statistical analyses

We used sampling weights to adjust for selection probabilities, oversampling, non-response, and differences between the sample and the entire US population. Given the right-skewed distribution of hs-CRP, we performed a natural logarithmic transformation on the hs-CRP values.Weighted univariate linear regression was employed to ascertain the correlations between sex steroid hormones and hs-CRP. We used weighted multivariable linear regression models to examine the relationship between sex hormones and hs-CRP, adjusting for various confounding factors. We performed trend tests to see if the effects of different ranges of sex hormones on hs-CRP were consistent. We adopted the Generalized Additive Model(GAM) to detect the non-linear association. If the relationship between sex hormones and hs-CRP was non-linear, a two-piecewise linear regression model was implemented to estimate the threshold effect of sex steroid hormones on hs-CRP. Subgroup analyses were conducted via stratified linear regression models, with the modification and interaction within subgroups assessed through the likelihood ratio test. These models were designed to explore associations between sex hormones and hs-CRP levels, rather than to infer causal relationships. Given the cross-sectional nature of the NHANES data, the causal direction of any observed associations cannot be determined. Therefore, we selected an analytical approach ideal for identifying correlations in cross-sectional data. We analyzed the data using the R software (version 4.2.2) and EmpowerStats (http://www.empowerstats.com). We considered a *P*-value < 0.05 as statistically significant.

## Results

### Baseline characteristics

The study population (**Table 1**), stratified by gender and age groups, exhibited significant disparities in levels of testosterone, estradiol, SHBG, age, and BMI (all *P*<0.001). Testosterone and estradiol levels were notably higher in adolescents, while SHBG was highest in children. BMI differed significantly between children and adolescents. Most participants were of normal weight (58.26%) and non-diabetic (99.38%).Importantly, pubertal status significantly differed between children and adolescents (*P*<0.001), with a noteworthy proportion of female children (21.85%) already in puberty. This finding prompted us to mainly categorize our population based on pubertal status for subsequent analysis.

**Table 1:** Characteristics of the population.

Characteristic	Total population	Total population ^a^	Male Children (6–11 years)	Male Adolescents (12–19 years)	Female Children (6–11 years)	Female Adolescents (12–19 years)	P-value
Testosterone (ng/dl, median, Q1-Q3)	18.25 (4.26-183.25)	24.00 (5.22-256.00)	3.62 (2.11-5.89)	387.00 (249.75-549.00)	4.62 (2.86-9.55)	24.50 (17.80-32.20)	<0.001
Estradiol (pg/ml, median,Q1-Q3)	12.35 (2.11-31.55)	15.00 (2.11-33.70)	2.11 (2.11-2.11)	19.40 (11.80-26.82)	2.11 (2.11-13.90)	57.10 (34.20-116.00)	<0.001
SHBG (nmol/l, median, Q1-Q3)	56.76 (34.41-95.55)	57.25 (34.77-93.93)	94.95 (62.72-134.88)	33.83 (22.85-47.23)	80.81 (49.66-112.70)	48.66 (33.41-74.41)	<0.001
Hs-CRP (mg/L, median, Q1-Q3)	0.40 (0.08-1.20)	0.30 (0.08-1.10)	0.30 (0.08-1.20)	0.40 (0.08-1.17)	0.30 (0.08-1.10)	0.40 (0.08-1.40)	0.668
Age (year, mean ± SD)	12.00 ± 3.86	12.48 ± 0.10	8.56 ± 1.69	15.35 ± 2.23	8.53 ± 1.72	14.98 ± 2.13	<0.001
PIR (median, Q1-Q3)	1.47 (0.65-2.75)	2.04 (0.98-3.52)	1.52 (0.73-2.99)	1.42 (0.61-2.68)	1.50 (0.58-2.67)	1.47 (0.74-2.64)	0.498
BMI (kg/m^2^, mean ± SD)	21.63 ± 5.80	21.77 ± 0.28	18.84 ± 4.06	24.00 ± 6.25	18.99 ± 4.11	24.33 ± 5.71	<0.001
BMI Category ^b^							0.150
Underweight (N,%)	45 (2.55%)	3.30%	8 (1.87%)	20 (3.89%)	10 (2.38%)	7 (1.73%)	
Normal Weight (N,%)	1030 (58.26%)	58.79%	257 (60.05%)	294 (57.20%)	257 (61.05%)	222 (54.81%)	
Overweight (N,%)	319 (18.04%)	18.05%	67 (15.65%)	89 (17.32%)	76 (18.05%)	87 (21.48%)	
Obese (N,%)	374 (21.15%)	19.86%	96 (22.43%)	111 (21.60%)	78 (18.53%)	89 (21.98%)	
TC (mg/dL, mean ± SD)	154.98 (27.63)	154.77 (0.95)	157.43 (25.20)	152.20 (28.49)	156.36 (27.08)	154.46 (29.29)	0.021
Race							0.438
Mexican American (N,%)	432 (24.43%)	16.68%	99 (23.13%)	119 (23.15%)	111 (26.37%)	103 (25.43%)	
Other Hispanic (N,%)	248 (14.03%)	9.86%	65 (15.19%)	56 (10.89%)	66 (15.68%)	61 (15.06%)	
Non-Hispanic White (N,%)	445 (25.17%)	50.26%	104 (24.30%)	145 (28.21%)	103 (24.47%)	93 (22.96%)	
Non-Hispanic Black (N,%)	367 (20.76%)	12.80%	92 (21.50%)	112 (21.79%)	83 (19.71%)	80 (19.75%)	
Non-Hispanic Asian (N,%)	168 (9.50%)	5.02%	43 (10.05%)	54 (10.51%)	29 (6.89%)	42 (10.37%)	
Other Race (N,%)	108 (6.11%)	5.38%	25 (5.84%)	28 (5.45%)	29 (6.89%)	26 (6.42%)	
Education							<0.001
6th and below 6th grade (N,%)	1005 (56.84%)	50.99%	428 (100.00%)	79 (15.37%)	421 (100.00%)	77 (19.01%)	
Above 6th grade (N,%)	763 (43.16%)	49.01%	0 (0.00%)	435 (84.63%)	0 (0.00%)	328 (80.99%)	
Diabetes							0.753
Non-diabetes (N,%)	1757 (99.38%)	99.41%	426 (99.53%)	511 (99.42%)	419 (99.52%)	401 (99.01%)	
Diabetes (N,%)	11 (0.62%)	0.59%	2 (0.47%)	3 (0.58%)	2 (0.48%)	4 (0.99%)	
Session of blood sample collection							0.004
Morning (N,%)	738 (41.74%)	41.69%	154 (35.98%)	238 (46.30%)	161 (38.24%)	185 (45.68%)	
Afternoon (N,%)	663 (37.50%)	37.55%	168 (39.25%)	190 (36.96%)	162 (38.48%)	143 (35.31%)	
Evening (N,%)	367 (20.76%)	20.76%	106 (24.77%)	86 (16.73%)	98 (23.28%)	77 (19.01%)	
Physical activity (hour / week) ^c^							<0.001
Non-activity (N,%)	1385 (78.34%)	71.92%	428 (100.00%)	264 (51.36%)	421 (100.00%)	272 (67.16%)	
0.1-0.9 (N,%)	45 (2.55%)	2.99%	0 (0.00%)	29 (5.64%)	0 (0.00%)	16 (3.95%)	
1.0-3.4 (N,%)	122 (6.90%)	9.44%	0 (0.00%)	65 (12.65%)	0 (0.00%)	57 (14.07%)	
3.5-5.9 (N,%)	58 (3.28%)	4.54%	0 (0.00%)	41 (7.98%)	0 (0.00%)	17 (4.20%)	
≥6 (N,%)	158 (8.94%)	11.12%	0 (0.00%)	115 (22.37%)	0 (0.00%)	43 (10.62%)	
Puberty Status ^d^							<0.001
Pubertal (N,%)	1000 (56.56%)	61.84%	30 (7.01%)	482 (93.77%)	92 (21.85%)	396 (97.78%)	
Prepubertal (N,%)	768 (43.44%)	38.16%	398 (92.99%)	32 (6.23%)	329 (78.15%)	9 (2.22%)	

a Applied sampling weights. In this column, values following the mean denote Standard Error (SE), not Standard Deviation (SD);

b Underweight (BMI < 5th percentile), Normal weight (BMI 5th to < 85th percentiles), Overweight (BMI 85th to < 95th percentiles), Obese (BMI ≥ 95th percentile);

c Physical activity pertains to individuals aged 12 and above;

d Puberty status was defined as “pubertal” if testosterone ≥ 50 ng/dL in males, estradiol ≥ 20 pg/ml or menstrual period started in females, otherwise puberty status was defined as “prepubertal”;

SHBG, sex hormone-binding globulin; Hs-CRP, High-Sensitivity C-Reactive Protein; PIR, Poverty income ratio; BMI, body mass index; TC, Total Cholesterol

### Weighted univariate analysis

The **Supplementary Table 1** analysis revealed that testosterone, estradiol, SHBG, age, race, BMI, diabetes status, and examination time were significantly associated with hs-CRP levels across prepubertal and pubertal males and females. Notably, the association strength did not significantly differ between groups, barring estradiol, race, examination time, and BMI.

### Association between testosterone and high-sensitivity C-reactive protein

**Figure 2:**
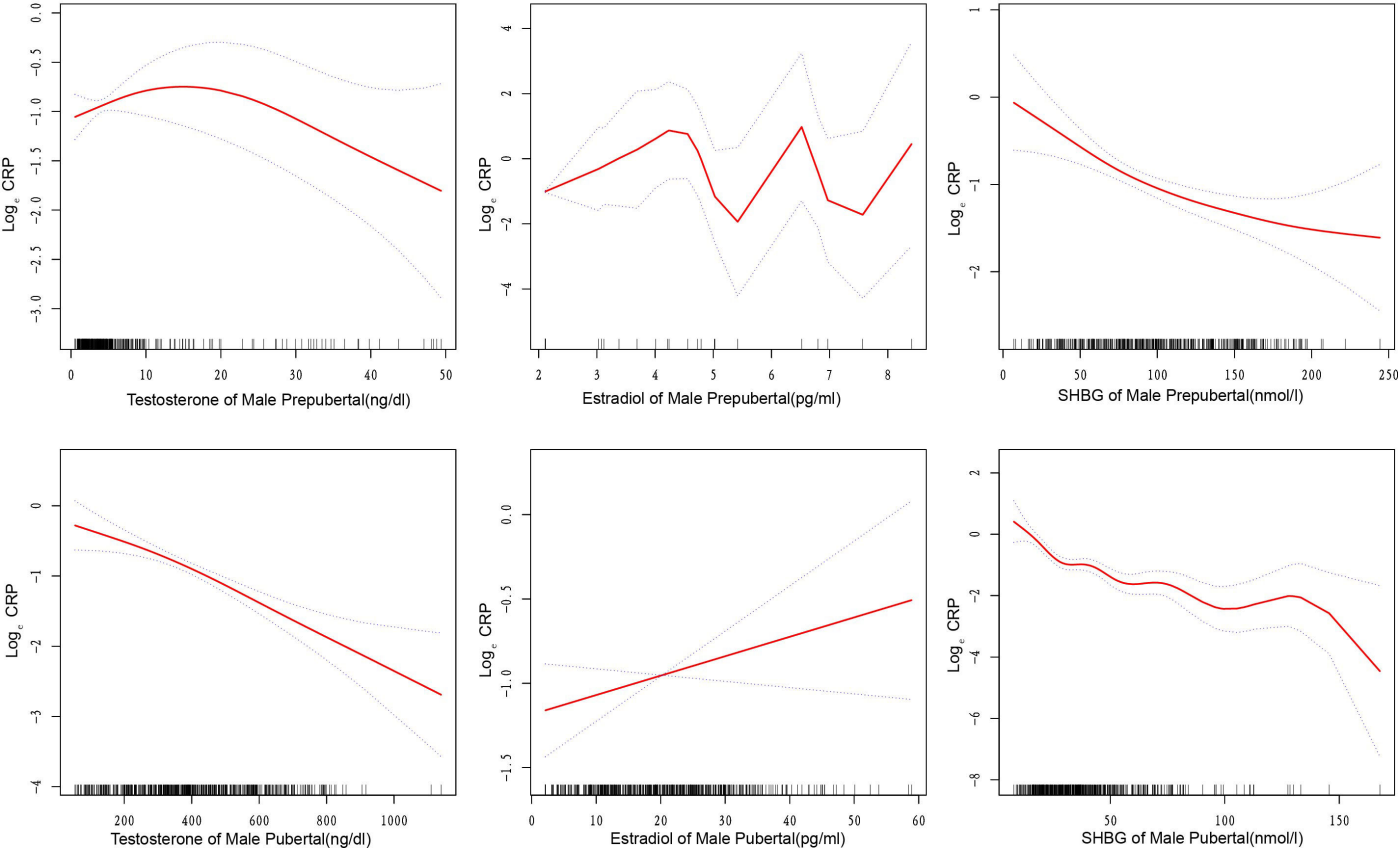
Curve fitting relationships between sex steroid hormones and high-sensitivity C-reactive protein in male children and adolescents. The solid and dashed lines in the graph represent the estimated values and corresponding 95% confidence intervals of high-sensitivity C-reactive protein, respectively. SHBG, sex hormone-binding globulin; Curve adjusted for age, race, education level, poverty income ratio, diabetes status, sample collection session, and total cholesterol.

In Male Prepubertal subjects, testosterone exhibited no significant link with hs-CRP (**Table 2**). A threshold effect was evident at a testosterone level of 8.90 ng/dl (**Supplementary Table 2**), associating positively below(β=0.082, *P*=0.047) and negatively above(β=-0.028, *P*=0.023) this threshold (**Figure 2**). Stratified analysis (**Supplementary Table 3**) revealed significant negative correlations in Mexican American(β=-0.027, *P*<0.05) and Other Hispanic groups(β=-0.044, *P*<0.05), while the Other Race group showed a positive correlation(β=0.073, *P*<0.05).

**Table 2:** Association between sex steroid hormones and high-sensitivity C-reactive protein.

Sex steroid hormones	Non-adjusted model β, 95%CI	Minimally-adjusted model β, 95%CI	Fully-adjusted model β, 95%CI
Testosterone (Male Prepubertal)	0.011 (-0.006, 0.027)	-0.012 (-0.032, 0.008)	-0.012 (-0.033, 0.008)
Q1(0.53 -4.24 ng/dl)	Reference	Reference	Reference
Q2(4.27 -18.20 ng/dl)	0.277 (-0.031, 0.584)	-0.002 (-0.355, 0.351)	-0.006 (-0.363, 0.350)
Q3(18.30 -181.00 ng/dl)	0.270 (-0.273, 0.812)	-0.372 (-1.014, 0.271)	-0.392 (-1.045, 0.261)
Q4(190.00 -1140.00 ng/dl)	0.236 (-0.268, 0.622)	-0.492 (-1.312, 0.618)	-0.218 (-1.362, 0.136)
P for trend	0.270	0.188	0.237
Testosterone (Male Pubertal)	-0.001 (-0.002, -0.000)	-0.002 (-0.002, -0.001)	-0.002 (-0.003, -0.001)
Q1(0.53 -4.24 ng/dl)	Reference	Reference	Reference
Q2(4.27 -18.20 ng/dl)	-0.000 (-0.027, 0.027)	-0.002 (-0.128, -0.001)	-0.016 (-0.032, -0.002)
Q3(18.30 -181.00 ng/dl)	-0.009 (-0.011, 0.030)	-0.017 (-0.006, 0.039)	-0.022 (-0.062, 0.008)
Q4(190.00 -1140.00 ng/dl)	0.193 (-0.159, 0.544)	-0.004 (-0.388, 0.379)	-0.051 (-0.456, 0.354)
P for trend	0.028	0.024	<0.001
Testosterone (Female Prepubertal)	0.055 (0.016, 0.093)	0.045 (-0.000, 0.091)	0.047 (0.001, 0.093)
Q1(0.53 -4.24 ng/dl)	Reference	Reference	Reference
Q2(4.27 -18.20 ng/dl)	0.303 (-0.017, 0.624)	0.118 (-0.244, 0.480)	0.153 (-0.213, 0.520)
Q3(18.30 -181.00 ng/dl)	1.140 (0.033, 2.247)	1.084 (-0.092, 2.260)	1.010 (-0.184, 2.203)
Q4(190.00 -1140.00 ng/dl)	0.392 (-0.483, 0.461)	0.739 (-0.830, 0.949)	0.715 (-0.039, 2.491)
P for trend	<0.001	0.061	0.046
Testosterone (Female Pubertal)	0.008 (-0.002, 0.017)	0.004 (-0.005, 0.014)	0.002 (-0.007, 0.012)
Q1(0.53 -4.24 ng/dl)	Reference	Reference	Reference
Q2(4.27 -18.20 ng/dl)	1.519 (-1.297, 4.335)	2.056 (-0.682, 4.794)	1.792 (-0.916, 4.500)
Q3(18.30 -181.00 ng/dl)	1.495 (-1.317, 4.306)	1.948 (-0.777, 4.674)	1.672 (-1.027, 4.370)
Q4(190.00 -1140.00 ng/dl)	0.986 (-0.110, 3.062)	1.015 (-0.609, 3.039)	1.017 (-0.036, 3.070)
P for trend	0.312	0.171	0.659
Estradiol (Male Prepubertal)	0.261 (0.050, 0.472)	0.183 (-0.028, 0.395)	0.185 (-0.031, 0.401)
Q1(2.11-6.18 pg/ml)	Reference	Reference	Reference
Q2(6.20-12.30 pg/ml)	0.742 (-0.163, 1.648)	0.430 (-0.471, 1.330)	0.405 (-0.510, 1.320)
Q3(12.40-31.50 pg/ml)	0.167 (-0.189, 0.185)	0.178 (-0.205, 1.151)	0.175 (-0.232,1.117)
Q4(31.70-564.00 pg/ml)	-0.298 (-0.319, 0.276)	-0.310 (-0.333, 0.288)	-0.300 (-0.353, 0.247)
P for trend	0. 012	0.350	0.094
Estradiol (Male Pubertal)	0.016 (0.005, 0.028)	0.013 (-0.002, 0.027)	0.012 (-0.004, 0.027)
Q1(2.11-6.18 pg/ml)	Reference	Reference	Reference
Q2(6.20-12.30 pg/ml)	0.791 (-0.234, 1.349)	0.738 (-0.161, 1.315)	0.729 (-0.132, 1.326)
Q3(12.40-31.50 pg/ml)	0.991 (-0.431, 1.551)	0.923 (-0.294, 1.551)	0.936 (-0.279, 1.594)
Q4(31.70-564.00 pg/ml)	1.274 (-0.539, 2.010)	1.208 (-0.393, 2.024)	1.235 (-0.390, 2.080)
P for trend	0.001	0.088	0.089
Estradiol (Female Prepubertal)	0.037 (-0.005, 0.079)	0.007 (-0.041, 0.055)	0.016 (-0.033, 0.065)
Q1(2.11-6.18 pg/ml)	Reference	Reference	Reference
Q2(6.20-12.30 pg/ml)	0.284 (-0.093, 0.660)	-0.026 (-0.458, 0.406)	0.037 (-0.401, 0.476)
Q3(12.40-31.50 pg/ml)	0.167 (-0.189, 0.145)	-0.178 (-0.205, 0.151)	-0.175 (-0.232, 0.117)
Q4(31.70-564.00 pg/ml)	0.298 (-0.319, 0.276)	-0.310 (-0.333, 0.288)	-0.300 (-0.353, 0.247)
P for trend	0.141	0.907	0.867
Estradiol (Female Pubertal)	-0.002 (-0.003, -0.000)	-0.003 (-0.004, -0.001)	-0.002 (-0.004, -0.001)
Q1(2.11-6.18 pg/ml)	Reference	Reference	Reference
Q2(6.20-12.30 pg/ml)	-0.094 (-0.119, 0.069)	-0.101 (-0.129, 0.074)	-0.099 (-0.160, 0.037)
Q3(12.40-31.50 pg/ml)	-0.142 (-0.734, 0.450)	0.000 (-0.573, 0.574)	-0.054 (-0.619, 0.512)
Q4(31.70-564.00 pg/ml)	-0.255 (-0.817, 0.307)	-0.207 (-0.750, 0.337)	-0.297 (-0.837, 0.243)
P for trend	0.027	0.034	0.033
SHBG(Male Prepubertal)	-0.016 (-0.019, -0.013)	-0.015 (-0.018, -0.012)	-0.015 (-0.018, -0.012)
Q1(4.81-34.31 nmol/l)	Reference	Reference	Reference
Q2(34.44-56.51 nmol/l)	-0.654 (-1.226, -0.082)	-0.627 (-1.200, -0.054)	-0.611 (-1.190, -0.032)
Q3(56.76-95.54 nmol/l)	-1.776 (-2.285, -1.266)	-1.697 (-2.222, -1.172)	-1.707 (-2.240, -1.175)
Q4(95.57-293.20 nmol/l)	-2.475 (-2.962, -1.988)	-2.365 (-2.879, -1.850)	-2.393 (-2.916, -1.870)
P for trend	<0.001	0.013	0.013
SHBG(Male Pubertal)	-0.021 (-0.026, -0.016)	-0.022 (-0.028, -0.017)	-0.023 (-0.029, -0.017)
Q1(4.81-34.31 nmol/l)	Reference	Reference	Reference
Q2(34.44-56.51 nmol/l)	-0.448 (-0.707, -0.189)	-0.439 (-0.707, -0.171)	-0.438 (-0.705, -0.170)
Q3(56.76-95.54 nmol/l)	-0.974 (-1.335, -0.613)	-0.970 (-1.363, -0.577)	-0.943 (-1.341, -0.544)
Q4(95.57-293.20 nmol/l)	-1.576 (-2.252, -0.901)	-1.609 (-2.323, -0.895)	-1.669 (-2.391, -0.947)
P for trend	<0.001	0.041	0.020
SHBG(Female Prepubertal)	-0.015 (-0.018, -0.012)	-0.014 (-0.017, -0.011)	-0.014 (-0.018, -0.011)
Q1(4.81-34.31 nmol/l)	Reference	Reference	Reference
Q2(34.44-56.51 nmol/l)	-0.477 (-1.075, 0.120)	-0.410 (-1.016, 0.195)	-0.445 (-1.057, 0.168)
Q3(56.76-95.54 nmol/l)	-1.334 (-1.898, -0.769)	-1.210 (-1.786, -0.634)	-1.213 (-1.797, -0.629)
Q4(95.57-293.20 nmol/l)	-1.929 (-2.474, -1.384)	-1.792 (-2.359, -1.225)	-1.823 (-2.395, -1.251)
P for trend	<0.001	0.011	<0.001
SHBG(Female Pubertal)	-0.013 (-0.017, -0.010)	-0.013 (-0.016, -0.010)	-0.013 (-0.016, -0.009)
Q1(4.81-34.31 nmol/l)	Reference	Reference	Reference
Q2(34.44-56.51 nmol/l)	-0.763 (-1.080, -0.446)	-0.672 (-0.982, -0.362)	-0.551 (-0.863, -0.239)
Q3(56.76-95.54 nmol/l)	-1.175 (-1.494, -0.857)	-1.095 (-1.408, -0.782)	-1.029 (-1.340, -0.717)
Q4(95.57-293.20 nmol/l)	-1.559 (-1.974, -1.144)	-1.472 (-1.885, -1.060)	-1.434 (-1.844, -1.025)
P for trend	<0.001	0.019	0.021

SHBG, sex hormone-binding globulin; The number of participants in each quartile group (Q1-Q4) is 442 individuals;

Non-adjusted model: no covariates were adjusted; Minimally-adjusted model: adjusted for age and race;

Fully-adjusted model: adjusted for age, race, education level, poverty income ratio, diabetes status, session of blood sample collection, total cholesterol. All models were weighted.

In Male Pubertal subjects, a consistent negative association was evident between testosterone and hs-CRP levels (β=-0.002, P<0.05, **Table 2**). **Supplementary Table 2** revealed a threshold effect at 224.00 ng/dl, where above this level, the association was more pronounced(β=-0.003, P<0.001) (**Figure 2**). The results of the stratified analysis (**Supplementary Table 3**) also support the negative correlation between testosterone and hs-CRP.

For Female Prepubertal subjects, a significant positive association was found between testosterone and hs-CRP in the fully-adjusted model in **Table 2** (β=0.047, *P*<0.05). However, the piecewise linear regression model in **Supplementary Table 2** failed to detect any significant threshold effect (**Supplementary Figure 1**). In the stratified analysis for the Female Prepubertal group (**Supplementary Table 3**), a significant negative relationship is demonstrated in overweight children (β=-0.077, *P*<0.05).

For Female Pubertal subjects, no significant association between testosterone and CRP was found in all models in **Table 2**, and likewise, no significant threshold effect was detected in **Supplementary Table 2** and **Supplementary Figure 1**. In the stratified analysis of female pubertal development, a significant negative association was observed among Mexican American children(β=-0.016, *P*<0.05) and those engaging in over 6 hours of physical activity per week(β=-0.048, *P*<0.05). Conversely, a significant positive relationship was found in Non-Hispanic Asian children (β=0.023, *P*<0.05).

### Association between estradiol and high-sensitivity C-reactive protein

In conducting multivariate regression analysis (**Table 2**) and threshold effect analysis (**Supplementary Table 2**), we did not identify significant associations between estradiol and hs-CRP in Male Prepubertal, Male Pubertal, and Female Prepubertal cohorts (**Figure 2**; **Supplementary Figure 1**). However, upon conducting stratified analyses within these three populations, we identified several subgroups where the relationship between estradiol and hs-CRP exhibited statistically significant associations. These included both positive and negative correlations (**Supplementary Table 4**).

In the Female Pubertal group, a significant negative correlation was observed between estradiol and hs-CRP(β=-0.002, *P*<0.05), and this inverse relationship was more pronounced when estradiol was less than or equal to 183 pg/ml(β=-0.004, *P*=0.001). In the Female Pubertal group, a significant positive correlation was observed between estradiol and hs-CRP when physical activity was between 3.5-5.9 hours per week(β=0.017, *P*<0.05, **Supplementary Table 4**).

### Association between SHBG and high-sensitivity C-reactive protein

From **Table 2**, for each subgroup, there exists a significant negative association between SHBG and hs-CRP, with the relationship holding across various models adjusting for different sets of covariates(all *P*<0.05). For the Male Prepubertal, Male Pubertal, Female Prepubertal, and Female Pubertal groups, the β coefficients suggest a stronger negative correlation below the respective inflection points of 72.09, 25.74, 149.10, and 56.48 nmol/l (**Supplementary Table 2**, **Figure 2**, and **Supplementary Figure 1**).

The results from stratified analysis also support a negative correlation between SHBG levels and hs-CRP (**Supplementary Table 5**). However, a notable exception is observed in the ‘Above 6th grade’ group within the Female Prepubertal population, where a positive correlation is identified(β=0.035, *P*<0.05).

## Discussion

Our research uncovers a distinct gender and age-related difference in the relationship between testosterone and hs-CRP. In Male Prepubertal group, an initial increase in testosterone levels leads to a transient surge in hs-CRP (β=0.082, *P*=0.047), subsequently resulting in an overall decline (β=-0.028, *P*=0.023). Contrarily, in Male Pubertal, hs-CRP levels consistently decrease under the influence of testosterone (β=-0.002, *P*<0.05). In Female Prepubertal, a significant positive correlation between testosterone and hs-CRP is observed (β=0.047, *P*<0.05). However, no significant correlation is found in Female Pubertal (β=0.002, *P*>0.05). Previous studies commonly support the negative correlation between testosterone and CRP in both adolescent (10) and adult males (5, 17), to some extent, validating the hypothesis that testosterone has anti-inflammatory properties in males. In females, the relationship between testosterone and CRP appears diverse, even contrary. The research conducted by de Dios O and colleagues did not identify any correlation between testosterone and hs-CRP in female adolescents aged between 12 and 16 years (10). In postmenopausal women, some studies have reported a positive correlation between serum testosterone levels and CRP (8). However, there are also studies concluding an inverse relationship between serum testosterone levels and CRP in postmenopausal women (9, 18). The level of testosterone plays a crucial role in modulating inflammatory processes, which is achieved by suppressing the expansion, differentiation, and function of adipocytes, curtailing the formation of cytokines (leptin, IL-6, TNF-α, MCP-1, resistin), and concurrently promoting the secretion of adiponectin (4). Future research is necessary to explore the interaction mechanisms between testosterone and hs-CRP.

Our study found a negative correlation between estradiol and hs-CRP (β=-0.002, *P*<0.05) solely within the Female Pubertal population. No such correlation was observed in the Male Prepubertal, Male Pubertal, and Female Prepubertal groups. Previous studies on adult females have indicated that the relationship between estradiol and CRP is negative pre-menopause (7, 19), but turns positive post-menopause (8, 18). This suggests a potentially complex and dynamic relationship between these variables across different stages of life. Throughout the inflammatory reaction, pro-inflammatory cytokines prompt the synthesis of NO in cells like monocytes, macrophages, and neutrophils. During the process of phagocytosis, NO, when discharged by tissue macrophages, operates as a positive feedback entity, catalyzing the attraction of additional phagocytes. Physiological levels of estrogen maintained the nCRP-facilitated decrease in NO production in LPS-stimulated monocytes, concurrently, estrogen countered the mCRP-induced increase of NO production in the same cells (20). This aligns with earlier research demonstrating that estrogen attenuates pro-inflammatory responses, which includes the production of NO and inflammatory cytokines.

Our study reveals a consistent negative correlation between SHBG and hs-CRP in both children and adolescents. This finding is not isolated, as it aligns with the results of prior research. Such negative correlation between SHBG and C-reactive protein has been confirmed in various populations, including adolescents (10), adult males (5), premenopausal women (19), and postmenopausal women (9). SHBG, secreted by the liver into the bloodstream, avidly binds to both androgens and estrogens, thereby regulating their bioavailability. BMI has been traditionally considered a primary determinant of circulating SHBG concentration, with a reported consistent negative correlation between BMI and plasma SHBG levels (21). Lower serum SHBG concentrations in overweight individuals serve as a biomarker of metabolic syndrome (22) and indicate an increased risk of type 2 diabetes(T2D) (23) and cardiovascular diseases(CVD) (24). Obesity-related endocrine mechanisms and chronic inflammation are associated with the reduction of SHBG prior to puberty, suggesting that lower SHBG levels could indicate an earlier onset of puberty (25). Reviews have proposed that the subtle inflammation and changes in pro-inflammatory/anti-inflammatory cytokines (i.e., TNFα, IL-1β, and adiponectin) occurring in obesity and T2D may be the primary cause of decreased SHBG levels, rather than hyperinsulinemia (24). It remains to be elucidated whether the decrease in SHBG is merely a biomarker, or if it actively participates in the inflammatory response process related to CRP, and subsequently contributes to the pathogenesis of obesity, T2D, fatty liver, and cardiovascular diseases.

Our study possesses several strengths. First, we have filled a research gap in exploring the relationship between sex steroid hormones and hs-CRP in children populations(6-11 years). Additionally, we categorized children and adolescents into prepubertal and pubertal groups based on hormone levels, offering a more reliable demographic foundation for researching the association between sex steroid hormones and hs-CRP. Furthermore, the data utilized in our study was obtained from the NHANES database, ensuring the accuracy of serum hormone and hs-CRP measurements. We also weighted our data to ensure that our final results would be representative of the overall health status of children and adolescents in the United States.

Nevertheless, there are some limitations in our study. Although we conducted detailed stratified analyses for various covariates, the unequal distribution of sample sizes across certain groups may have resulted in insufficient statistical power for some analyses. For instance, in the stratified analysis of testosterone and high-sensitivity C-reactive protein (**Supplementary Table 3**), the Normal Weight group included 1,030 participants, while the Underweight group comprised only 45 participants. Therefore, the interpretation of these results must take into account the limitations posed by the small sample sizes. Even though we adjusted for potential confounders, there might still be unadjusted factors that could influence our results. The lack of data on gonadotropin-releasing hormone, gonadotropins, and crucial enzymes involved in hormone responses restricted us from further delving into the underlying biological mechanisms. Importantly, it should be noted that our study is cross-sectional, thereby preventing us from establishing a causal relationship between sex steroid hormones and hs-CRP. This design captures data at a single point in time, limiting our ability to track changes in variables over time or to ascertain causal sequences. Future research should employ longitudinal designs to overcome these limitations and validate our findings.

## Conclusions

Our findings indicate that the association between sex steroid hormones and high-sensitivity C-reactive protein (hs-CRP) levels among American children and adolescents is conditional and influenced by multiple factors. Specifically, this association is influenced by several factors including age, body mass index (BMI), pubertal status, hormone levels, among others. For instance, in prepubertal males, testosterone levels below 8.90 ng/dL positively correlate with hs-CRP levels, while levels above this threshold show a negative correlation. This indicates that the association between sex steroid hormones and inflammatory markers involves a multifaceted interplay, potentially including other biomarkers and environmental factors not assessed in this study. Further research is needed to explore these dimensions to better elucidate the correlations between sex hormones and inflammatory markers.

## Data Availability

The original contributions presented in the study are included in the article/**Supplementary Material**. Further inquiries can be directed to the corresponding author.

## Supplementary material

Supplementary Figure 1 Curve fitting relationships between sex steroid hormones and high-sensitivity C-reactive protein in female children and adolescents. The solid and dashed lines in the graph represent the estimated values and corresponding 95% confidence intervals of high-sensitivity C-reactive protein, respectively. SHBG: sex hormone-binding globulin; Curve adjusted for age, race, education level, poverty income ratio, diabetes status, sample collection session, and total cholesterol.
