# Supplementary material for: Conditional associations of sex steroid hormones with C-reactive protein levels in American children and adolescents: evidence from NHANES 2015-2016
Source: Front Endocrinol (Lausanne). 2024 Sep 24;15:1431984. doi: 10.3389/fendo.2024.1431984 (PMC11458447; doi:10.3389/fendo.2024.1431984)
Supplement: Supplementary Figure 1 — Curve fitting relationships between sex steroid hormones and high-sensitivity C-reactive protein in female children and adolescents. The solid and dashed lines in the graph represent the estimated values and corresponding 95% confidence intervals of high-sensitivity C-reactive protein, respectively. SHBG: sex hormone-binding globulin; Curve adjusted for age, race, education level, poverty income ratio, diabetes status, sample collection session, and total cholesterol. [file DataSheet1.docx]

**Supplementary Materials**

**
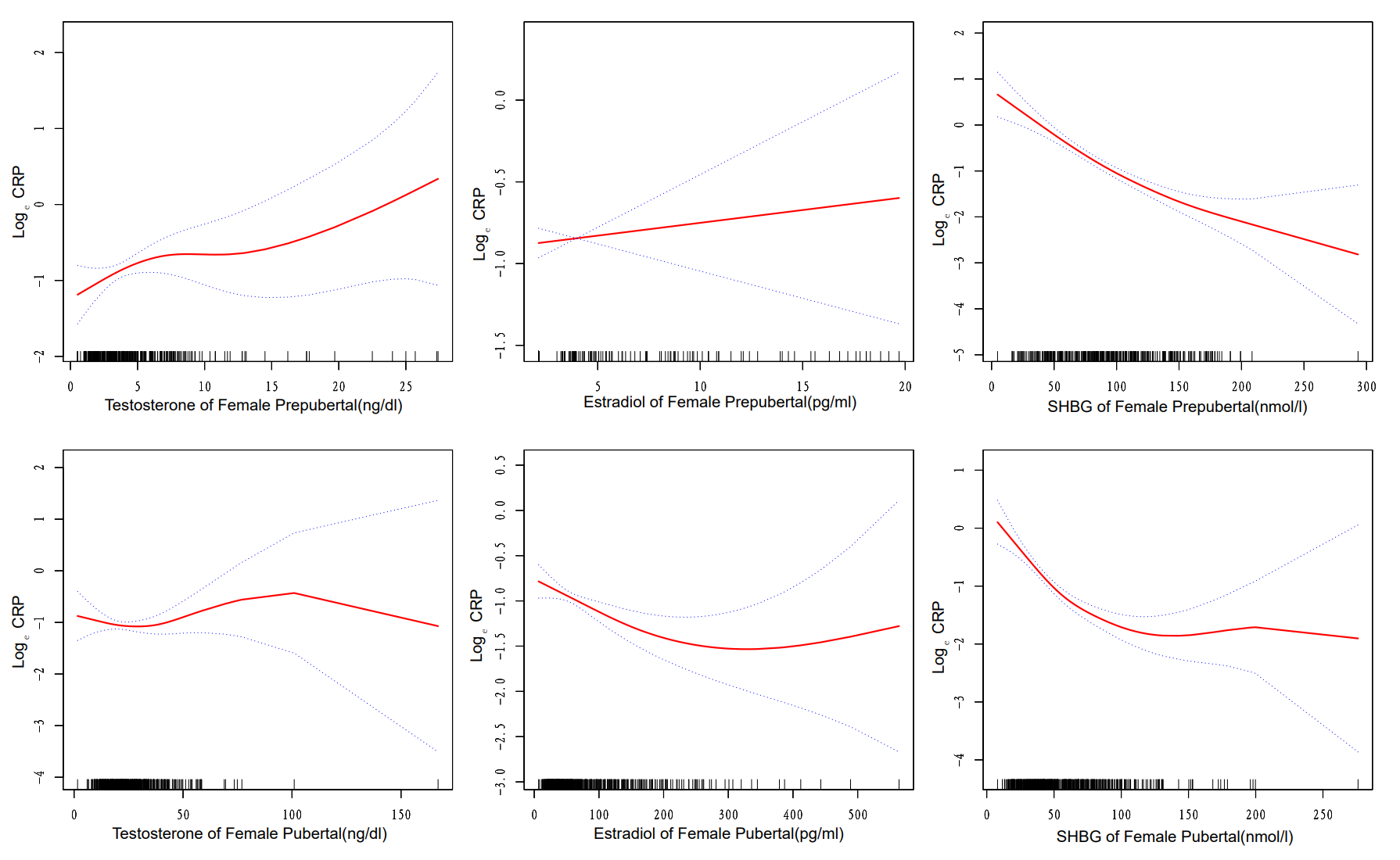
**

**Supplementary Figure 1: Curve fitting relationships between sex steroid hormones and high-sensitivity C-reactive protein in female children and adolescents.** The solid and dashed lines in the graph represent the estimated values and corresponding 95% confidence intervals of high-sensitivity C-reactive protein, respectively. SHBG: sex hormone‑binding globulin; Curve adjusted for age, race, education level, poverty income ratio, diabetes status, sample collection session, and total cholesterol.

**Supplementary Table 1-5**

**Supplementary Table 1: Weighted univariate analysis for high-sensitivity C-reactive protein**

| Population | Male Prepubertal^a^ | Male Pubertal | Female Prepubertal | Female Pubertal | P-interaction |
| --- | --- | --- | --- | --- | --- |
| Testosterone | 0.011 (-0.006, 0.027) | -0.001 (-0.002, -0.000) * | 0.055 (0.016, 0.093) * | 0.008 (-0.002, 0.017) | 0.3617 |
| Estradiol | 0.261 (0.050, 0.472) * | 0.016 (0.005, 0.028) * | 0.037 (-0.005, 0.079) | -0.002 (-0.003, -0.000) * | 0.0007 |
| SHBG | -0.016 (-0.019, -0.013) ** | -0.021 (-0.026, -0.016) ** | -0.015 (-0.018, -0.012) ** | -0.013 (-0.017, -0.010) ** | 0.0701 |
| Age | 0.131 (0.020, 0.242) * | 0.069 (0.005, 0.133) * | 0.163 (0.034, 0.293) * | 0.125 (0.069, 0.181) * | 0.4728 |
| Race |  |  |  |  | <0.0001 |
| Mexican American | Reference | Reference | Reference | Reference |  |
| Other Hispanic | 0.915 (0.446, 1.385) * | 1.042 (0.074, 2.009) * | 1.193 (0.562, 1.824) * | 0.788 (0.297, 1.279) * |  |
| Non-Hispanic White | 0.580 (-0.101, 1.262) | 0.553 (0.029, 1.077) * | 0.556 (0.111, 1.001) * | 0.736 (0.301, 1.170) * |  |
| Non-Hispanic Black | 0.510 (-0.035, 1.055) | 0.844 (0.297, 1.391) * | 0.880 (0.406, 1.355) * | 0.624 (0.238, 1.009) * |  |
| Non-Hispanic Asian | 0.909 (0.401, 1.417) * | 0.625 (0.154, 1.095) * | 1.093 (0.105, 2.081) * | 0.722 (0.184, 1.260) * |  |
| Other Race | 0.668 (0.038, 1.298) * | 0.766 (-0.172, 1.705) | 1.139 (0.174, 2.104) * | 0.818 (-0.316, 1.816) |  |
| Education |  |  |  |  | 0.4470 |
| 6th and below 6th grade | Reference | Reference | Reference | Reference |  |
| Above 6th grade | 0.498 (-0.713, 1.709) | 0.474 (-0.005, 0.953) | 1.323 (-0.153, 2.799) | 0.270 (-0.061, 0.600) |  |
| PIR | -0.023 (-0.145, 0.099) | -0.149 (-0.216, -0.082) * | -0.199 (-0.312, -0.087) * | -0.112 (-0.184, -0.040) * | 0.2150 |
| BMI | 0.207 (0.182, 0.233) ** | 0.129 (0.112, 0.145) ** | 0.195 (0.166, 0.225) ** | 0.142 (0.121, 0.164) ** | <0.0001 |
| BMI Category ^b^ |  |  |  |  | <0.0001 |
| Underweight | Reference | Reference | Reference | Reference |  |
| NormalWeight | -1.852 (-2.114, -1.590) * | -1.774 (-2.061, -1.488) * | -1.752 (-2.083, -1.421) * | -1.890 (-2.173, -1.606) * |  |
| Overweight | -1.101 (-1.768, -0.434) * | -0.915 (-1.365, -0.465) * | -0.610 (-0.995, -0.226) * | -0.933 (-1.381, -0.484) * |  |
| Obese | 0.039 (-0.335, 0.413) | -0.109 (-0.487, 0.269) | 0.273 (-0.050, 0.596) | 0.368 (-0.037, 0.622) |  |
| Diabetes Status |  |  |  |  | 0.8867 |
| Non-diabetes | Reference | Reference | Reference | Reference |  |
| Diabetes | 1.880 (1.405, 2.356) ** | 1.420 (-0.449, 3.288) | 1.929 (1.741, 2.118) ** | 2.049 (1.314, 2.784) ** |  |
| Examine Time |  |  |  |  | 0.0001 |
| Morning | Reference | Reference | Reference | Reference |  |
| Afternoon | 0.093 (-0.288, 0.474) | -0.073 (-0.378, 0.231) | -0.006 (-0.314, 0.302) | -0.487 (-0.756, -0.217) * |  |
| Evening | -0.460 (-1.000, 0.080) | 0.087 (-0.226, 0.400) | 0.066 (-0.427, 0.559) | -0.034 (-0.391, 0.323) |  |
| Total Cholesterol | 0.006 (-0.001, 0.012) | 0.004 (0.000, 0.007) * | 0.001 (-0.004, 0.006) | 0.004 (0.001, 0.007) * | 0.3641 |

^a^ Puberty status was defined as “pubertal” if testosterone ≥ 50 ng/dL in males, estradiol ≥ 20 pg/ml or menstrual period started in females, otherwise puberty status was defined as “prepubertal” ;

^b^ Underweight (BMI < 5th percentile), Normal weight (BMI 5th to < 85th percentiles), Overweight (BMI 85th to < 95th percentiles), Obese (BMI ≥ 95th percentile);

**P-value <0.001,*P-value <0.05; SHBG: sex hormone-binding globulin; PIR: Poverty income ratio; BMI: body mass index;

**Supplementary Table 2 : Threshold effect analysis of sex steroid hormones on high-sensitivity C-reactive protein using piecewise linear regression models**

| Exposure | Inflection point (cm) | Group | β (95% CI) P | P for log likelihood ratio test |
| --- | --- | --- | --- | --- |
| Testosterone | 8.90 | ≤8.90 | 0.082 (0.002, 0.162) 0.047 | 0.016 |
| (Male Prepubertal)^a^ |  | >8.90 | -0.028 (-0.052, -0.004) 0.023 |  |
| Testosterone | 224.00 | ≤224.00 | 0.002 (-0.002, 0.005) 0.380 | 0.033 |
| (Male Pubertal) |  | >224.00 | -0.003 (-0.003, -0.002) <0.001 |  |
| Testosterone | 3.83 | ≤3.83 | 0.187 (-0.010, 0.384) 0.064 | 0.143 |
| (Female Prepubertal) |  | >3.83 | 0.031 (-0.020, 0.082) 0.240 |  |
| Testosterone | 17.50 | ≤17.50 | -0.045 (-0.100, 0.009) 0.104 | 0.079 |
| (Female Pubertal) |  | >17.50 | 0.006 (-0.004, 0.017) 0.253 |  |
| Estradiol | 4.30 | ≤4.30 | 0.577 (0.106, 1.048) 0.017 | 0.062 |
| (Male Prepubertal) |  | >4.30 | -0.314 (-0.889, 0.261) 0.285 |  |
| Estradiol | 5.26 | ≤5.26 | 0.216 (-0.031, 0.464) 0.087 | 0.099 |
| (Male Pubertal) |  | >5.26 | 0.009 (-0.007, 0.024) 0.280 |  |
| Estradiol | 3.38 | ≤3.38 | 0.101 (-0.268, 0.469) 0.593 | 0.641 |
| (Female Prepubertal) |  | >3.38 | 0.006 (-0.057, 0.070) 0.849 |  |
| Estradiol | 183.00 | ≤183.00 | -0.004 (-0.007, -0.002) 0.001 | 0.045 |
| (Female Pubertal) |  | >183.00 | 0.001 (-0.003, 0.005) 0.548 |  |
| SHBG | 72.09 | ≤72.09 | -0.020 (-0.032, -0.008) 0.001 | 0.019 |
| (Male Prepubertal) |  | >72.09 | -0.005 (-0.008, -0.001) 0.018 |  |
| SHBG | 25.74 | ≤25.74 | -0.082 (-0.114, -0.050) <0.001 | <0.001 |
| (Male Pubertal) |  | >25.74 | -0.017 (-0.024, -0.011) <0.001 |  |
| SHBG | 149.10 | ≤149.10 | -0.017 (-0.021, -0.013) <0.001 | 0.018 |
| (Female Prepubertal) |  | >149.10 | 0.001 (-0.012, 0.015) 0.859 |  |
| SHBG | 56.48 | ≤56.48 | -0.034 (-0.043, -0.024) <0.001 | <0.001 |
| (Female Pubertal) |  | >56.48 | -0.005 (-0.009, 0.000) 0.072 |  |

^a^ Puberty status was defined as “pubertal” if testosterone ≥ 50 ng/dL in males, estradiol ≥ 20 pg/ml or menstrual period started in females, otherwise puberty status was defined as “prepubertal” ;

SHBG: sex hormone-binding globulin

**Supplementary Table 3: Stratified analysis of testosterone and high-sensitivity C-reactive protein**

| Covariate | N,% | | Male Prepubertal ^a^ β(95%CI) | *P* * | | Male Pubertal β(95%CI) | *P* * | | Female Prepubertal β(95%CI) | *P* * | | Female Pubertal β(95%CI) | *P* * | |
| --- | --- | --- | --- | --- | --- | --- | --- | --- | --- | --- | --- | --- | --- | --- |
| Race |  | |  | <0.001 | |  | 0.297 | |  | <0.001 | |  | <0.001 | |
| Mexican American(N,%) | 432 (16.68%) | | -0.027 (-0.043, -0.011) * | |  | -0.002 (-0.004, -0.001) * | |  | -0.085 (-0.218, 0.047) |  | | -0.016 (-0.032, -0.000) * | |  |
| Other Hispanic (N,%) | 248 (9.86%) | | -0.044 (-0.075, -0.012) * | |  | -0.003 (-0.005, -0.001) * | |  | -0.039 (-0.167, 0.088) |  | | 0.019 (-0.001, 0.039) | |  |
| Non-Hispanic White (N,%) | 445 (50.26%) | | -0.017 (-0.042, 0.008) |  | | -0.001 (-0.002, -0.000) * | |  | -0.001 (-0.079, 0.077) |  | | -0.005 (-0.026, 0.015) | |  |
| Non-Hispanic Black (N,%) | 367 (12.80%) | | 0.018 (-0.017, 0.052) |  | | -0.002 (-0.004, -0.001) * | |  | 0.077 (0.020, 0.135) * |  | | 0.004 (-0.013, 0.021) | |  |
| Non-Hispanic Asian (N,%) | 168 (5.02%) | | -0.016 (-0.078, 0.045) |  | | -0.002 (-0.003, -0.000) * | |  | -0.089 (-0.220, 0.042) |  | | 0.023 (0.001, 0.045) * | |  |
| Other Race (N,%) | 108 (5.38%) | | 0.073 (0.038, 0.108) * |  | | -0.001 (-0.006, 0.004) |  | | 0.035 (-0.199, 0.269) |  | | -0.008 (-0.034, 0.018) |  | |
| Education level |  | |  | 0.785 | |  | 0.725 | |  | 0.006 | |  | 0.084 | |
| 6th and below 6th grade (N,%) | 1005 (50.99%) | | -0.014 (-0.036, 0.009) |  | | -0.001 (-0.004, 0.001) |  | | 0.013 (-0.044, 0.070) |  | | -0.022 (-0.045, 0.001) |  | |
| Above 6th grade (N,%) | 763 (49.01%) | | -0.022 (-0.074, 0.029) |  | | -0.002 (-0.003, -0.001) * | |  | 0.097 (0.046, 0.147) * |  | | 0.002 (-0.012, 0.016) |  | |
| Session of blood sample collection | | | | 0.366 | |  | 0.001 | |  | 0.032 | |  | 0.254 | |
| Morning (N,%) | 738 (41.69%) | | -0.028 (-0.053, -0.004) * | |  | -0.002 (-0.003, -0.001) * | |  | 0.043 (-0.092, 0.178) |  | | 0.006 (-0.013, 0.025) |  | |
| Afternoon (N,%) | 663 (37.55%) | | -0.005 (-0.030, 0.020) |  | | -0.002 (-0.003, 0.000) |  | | 0.090 (0.031, 0.148) * |  | | -0.005 (-0.026, 0.016) |  | |
| Evening (N,%) | 367 (20.76%) | | -0.007 (-0.043, 0.029) |  | | 0.001 (-0.001, 0.003) |  | | -0.024 (-0.076, 0.028) |  | | -0.013 (-0.033, 0.007) |  | |
| BMI Category ^b^ | | |  | 0.033 | |  | 0.150 | |  | 0.466 | |  | 0.075 | |
| Underweight (N,%) | 45 (3.30%) | | 0.023 (-0.002, 0.049) |  | | 0.000 (-0.001, 0.001) |  | | 0.159 (-0.759, 1.076) |  | | -0.009 (-0.029, 0.012) |  | |
| Normal Weight (N,%) | 1030 (58.79%) | | -0.014 (-0.034, 0.006) |  | | -0.000 (-0.001, 0.001) |  | | -0.014 (-0.067, 0.039) |  | | -0.005 (-0.015, 0.005) |  | |
| Overweight (N,%) | 319 (18.05%) | | -0.019 (-0.058, 0.019) |  | | -0.001 (-0.004, 0.002) |  | | -0.077 (-0.145, -0.008) * | |  | 0.013 (-0.005, 0.031) |  | |
| Obese (N,%) | 374 (19.86%) | | 0.016 (-0.047, 0.079) |  | | -0.002 (-0.004, -0.000) * | |  | -0.026 (-0.073, 0.022) |  | | -0.008 (-0.017, 0.001) |  | |
| Physical activity (hour / week) ^c^ | | | |  | |  | 0.535 | |  |  | |  | 0.008 | |
| Non-activity (N,%) | | 1385(71.92%) | |  | | -0.001 (-0.002, 0.000) |  | |  |  | | 0.010 (-0.002, 0.022) |  | |
| 0.1-0.9 (N,%) | 45 (2.99%) | |  |  | | -0.002 (-0.004, 0.001) |  | |  |  | | -0.002 (-0.050, 0.046) |  | |
| 1.0-3.4 (N,%) | 122 (9.44%) | |  |  | | -0.002 (-0.005, -0.000) * | |  |  |  | | -0.012 (-0.030, 0.006) |  | |
| 3.5-5.9 (N,%) | 58 (4.54%) | |  |  | | -0.002 (-0.007, 0.002) | |  |  |  | | -0.027 (-0.122, 0.069) |  | |
| ≥6 (N,%) | 158 (11.12%) | | |  | | -0.002 (-0.005, -0.000) * | |  |  |  | | -0.048 (-0.092, -0.004) * | |  |

^a^ Puberty status was defined as “pubertal” if testosterone ≥ 50 ng/dL in males, estradiol ≥ 20 pg/ml or menstrual period started in females, otherwise puberty status was defined as “prepubertal” ;

^b^ Underweight (BMI < 5th percentile), Normal weight (BMI 5th to < 85th percentiles), Overweight (BMI 85th to < 95th percentiles), Obese (BMI ≥ 95th percentile);

^c^ Physical activity pertains to individuals aged 12 and above; *P* * *P* for interaction; BMI: body mass index; * *P*-value <0.05;

**Supplementary Table 4: Stratified analysis of estradiol and high-sensitivity C-reactive protein**

| Covariate | N,% | | Male Prepubertal ^a^ β(95%CI) | *P* * | | Male Pubertal β(95%CI) | *P* * | Female Prepubertal β(95%CI) | *P* * | | Female Pubertal β(95%CI) | *P* * | | |
| --- | --- | --- | --- | --- | --- | --- | --- | --- | --- | --- | --- | --- | --- | --- |
| Race |  | |  | <0.001 | |  | 0.001 |  | 0.038 | |  | 0.413 | | |
| Mexican American(N,%) | 432 (16.68%) | | -0.060 (-0.249, 0.128) |  | | 0.024 (-0.006, 0.055) |  | -0.064 (-0.138, 0.011) |  | | -0.002 (-0.005, 0.001) |  | | |
| Other Hispanic (N,%) | 248 (9.86%) | | 0.292 (0.079, 0.505) * |  | | -0.020 (-0.052, 0.011) |  | -0.054 (-0.231, 0.124) |  | | 0.000 (-0.003, 0.004) |  | | |
| Non-Hispanic White (N,%) | 445 (50.26%) | | 0.319 (-0.256, 0.893) |  | | 0.041 (0.013, 0.069) * |  | 0.055 (-0.017, 0.127) |  | | -0.006 (-0.010, -0.002) * | |  | |
| Non-Hispanic Black (N,%) | 367 (12.80%) | | 0.195 (-0.174, 0.564) |  | | 0.006 (-0.019, 0.030) |  | 0.081 (-0.029, 0.190) |  | | -0.002 (-0.004, 0.000) | |  | |
| Non-Hispanic Asian (N,%) | 168 (5.02%) | | 0.474 (-1.634, 2.582) |  | | 0.030 (-0.005, 0.065) |  | 0.041 (-0.097, 0.180) |  | | -0.007 (-0.013, -0.000) * | |  | |
| Other Race (N,%) | 108 (5.38%) | | 0.454 (0.135, 0.773) * |  | | 0.040 (-0.064, 0.143) |  | 0.251 (-0.081, 0.584) |  | | -0.000 (-0.004, 0.004) |  | | |
| Education level |  | |  | 0.490 | |  | 0.907 |  | <0.001 | |  | 0.692 | | |
| 6th and below 6th grade (N,%) | 1005 (50.99%) | | 0.314 (-0.027, 0.656) |  | | 0.021 (-0.045, 0.087) |  | 0.031 (-0.020, 0.082) |  | | -0.004 (-0.007, -0.000) * | | |  |
| Above 6th grade (N,%) | 763 (49.01%) | | 0.206 (-0.030, 0.441) |  | | 0.025 (0.006, 0.044) * |  | 0.342 (0.201, 0.483) * |  | | -0.003 (-0.005, -0.001) * | | |  |
| Session of blood sample collection | | | | 0.123 | |  | 0.538 |  | 0.013 | |  | 0.439 | | |
| Morning (N,%) | 738 (41.69%) | | -0.150 (-0.498, 0.198) |  | | 0.020 (-0.009, 0.049) |  | 0.076 (0.024, 0.129) * |  | | -0.003 (-0.005, -0.000) * | |  | |
| Afternoon (N,%) | 663 (37.55%) | | 0.337 (-0.005, 0.678) |  | | 0.024 (-0.003, 0.051) |  | 0.032 (-0.085, 0.149) |  | | -0.002 (-0.003, -0.000) * | |  | |
| Evening (N,%) | 367 (20.76%) | | 0.584 (-0.038, 1.205) |  | | 0.042 (0.013, 0.070) * |  | -0.033 (-0.104, 0.038) |  | | -0.006 (-0.012, -0.000) * | |  | |
| BMI Categories ^b^ | | |  | <0.001 | |  | 0.761 |  | 0.265 | |  | 0.572 | | |
| Underweight (N,%) | 45 (3.30%) | | 0.474 (0.070, 0.878) * |  | | 0.035 (-0.047, 0.116) |  | -1.156 (-1.569, -0.743) * | |  | -0.002 (-0.004, -0.000) * | |  | |
| Normal Weight (N,%) | 1030 (58.79%) | | -0.255 (-0.423, -0.087) * | |  | 0.004 (-0.016, 0.025) |  | 0.009 (-0.046, 0.065) |  | | -0.003 (-0.005, -0.001) * | |  | |
| Overweight (N,%) | 319 (18.05%) | | 1.490 (0.655, 2.326) * |  | | -0.005 (-0.038, 0.028) |  | 0.025 (-0.037, 0.086) |  | | -0.002 (-0.004, 0.000) |  | | |
| Obese (N,%) | 374 (19.86%) | | 0.199 (-0.020, 0.418) |  | | 0.015 (-0.013, 0.043) |  | -0.045 (-0.105, 0.015) |  | | -0.001 (-0.002, 0.001) |  | | |
| Physical activity (hour / week) ^c^ | | | |  | |  | 0.312 |  |  | |  | <0.001 | | |
| Non-activity (N,%) | | 1385(71.92%) | |  | | 0.020 (0.001, 0.040) * |  |  |  | | -0.003 (-0.005, -0.001) * | |  | |
| 0.1-0.9 (N,%) | 45 (2.99%) | |  |  | | -0.006 (-0.054, 0.042) |  |  |  | | -0.008 (-0.015, -0.001) * | |  | |
| 1.0-3.4 (N,%) | 122 (9.44%) | |  |  | | 0.041 (-0.007, 0.090) |  |  |  | | -0.005 (-0.009, -0.001) * | |  | |
| 3.5-5.9 (N,%) | 58 (4.54%) | |  |  | | 0.018 (-0.034, 0.069) |  |  |  | | 0.017 (0.011, 0.024) * | |  | |
| ≥6 (N,%) | 158 (11.12%) | | |  | | 0.029 (-0.000, 0.058) |  |  |  | | -0.007 (-0.012, -0.002) * | |  | |

^a^ Puberty status was defined as “pubertal” if testosterone ≥ 50 ng/dL in males, estradiol ≥ 20 pg/ml or menstrual period started in females, otherwise puberty status was defined as “prepubertal” ;

^b^ Underweight (BMI < 5th percentile), Normal weight (BMI 5th to < 85th percentiles), Overweight (BMI 85th to < 95th percentiles), Obese (BMI ≥ 95th percentile);

^c^ Physical activity pertains to individuals aged 12 and above; *P* * *P* for interaction; BMI: body mass index; * *P*-value <0.05;

In the subgroup analysis, the 'race', 'education level', and 'blood collection session' subgroups were adjusted for 'age', 'race', 'education level', 'ratio of family income to poverty', 'diabetes', 'blood collection session', and 'cholesterol', excluding the subgroup variable. The same adjustments were made for the 'BMI Categories' and 'physical activity' subgroups.

**Supplementary Table 5: Stratified analysis of sex hormone-binding globulin and high-sensitivity C-reactive protein**

| Covariate | N,% | | Male Prepubertal ^a^ β(95%CI) | *P* * | | | Male Pubertal β(95%CI) | *P* * | | | | | Female Prepubertal β(95%CI) | *P* * | | | Female Pubertal β(95%CI) | *P* * | | |
| --- | --- | --- | --- | --- | --- | --- | --- | --- | --- | --- | --- | --- | --- | --- | --- | --- | --- | --- | --- | --- |
| Race |  | |  | 0.550 | | |  | 0.928 | | | | |  | 0.005 | | |  | 0.256 | | |
| Mexican American(N,%) | 432 (16.68%) | | -0.018 (-0.025, -0.011) * | | |  | -0.030 (-0.048, -0.011) * | | | | |  | -0.012 (-0.018, -0.006) * | |  | | -0.020 (-0.030, -0.011) * | |  | |
| Other Hispanic (N,%) | 248 (9.86%) | | -0.017 (-0.022, -0.012) * | | |  | -0.020 (-0.032, -0.007) * | | | | |  | -0.022 (-0.030, -0.015) * | |  | | -0.013 (-0.024, -0.001) * | |  | |
| Non-Hispanic White (N,%) | 445 (50.26%) | | -0.017 (-0.023, -0.011) * | | |  | -0.021 (-0.028, -0.013) * | | | | |  | -0.013 (-0.017, -0.008) * | |  | | -0.013 (-0.017, -0.008) * | |  | |
| Non-Hispanic Black (N,%) | 367 (12.80%) | | -0.014 (-0.022, -0.006) * | | |  | -0.025 (-0.038, -0.012) * | | | | |  | -0.015 (-0.022, -0.007) * | |  | | -0.013 (-0.021, -0.005) * | |  | |
| Non-Hispanic Asian (N,%) | 168 (5.02%) | | -0.011 (-0.019, -0.003) * | | |  | -0.032 (-0.057, -0.007) * | | | | |  | -0.000 (-0.022, 0.021) |  | | | -0.007 (-0.019, 0.005) | |  | |
| Other Race (N,%) | 108 (5.38%) | | -0.019 (-0.027, -0.011) * | | |  | -0.028 (-0.105, 0.048) | | | | |  | -0.019 (-0.029, -0.010) * | |  | | -0.009 (-0.021, 0.004) | |  | |
| Education level |  | |  | 0.022 | | |  | 0.231 | | | | |  | <0.001 | | |  | 0.681 | | |
| 6th and below 6th grade (N,%) | 1005 (50.99%) | | -0.016 (-0.020, -0.012) * | | |  | -0.016 (-0.025, -0.008) * | |  | | | | -0.014 (-0.018, -0.010) * | |  | | -0.012 (-0.017, -0.006) * | |  | |
| Above 6th grade (N,%) | 763 (49.01%) | | -0.027 (-0.036, -0.019) * | | |  | -0.025 (-0.032, -0.017) * | |  | | | | 0.035 (0.027, 0.043) * |  | | | -0.013 (-0.018, -0.008) * | |  | |
| Session of blood sample collection | | | | 0.021 | | |  | 0.273 | | | | |  | 0.333 | | |  | 0.781 | | |
| Morning (N,%) | 738 (41.69%) | | -0.016 (-0.022, -0.010) * | |  | | -0.022 (-0.030, -0.014) * | | | |  | | -0.017 (-0.023, -0.010) * | | |  | -0.012 (-0.016, -0.008) * | |  | |
| Afternoon (N,%) | 663 (37.55%) | | -0.019 (-0.024, -0.014) * | |  | | -0.020 (-0.028, -0.012) * | | | |  | | -0.012 (-0.015, -0.009) * | | |  | -0.012 (-0.019, -0.005) * | |  | |
| Evening (N,%) | 367 (20.76%) | | -0.013 (-0.018, -0.008) * | |  | | -0.027 (-0.036, -0.018) * | |  | | | | -0.012 (-0.021, -0.004) * | | |  | -0.017 (-0.030, -0.003) * | |  | |
| BMI Categories ^b^ | | |  | <0.001 | | |  | 0.344 | | | | |  | 0.846 | | |  | 0.202 | | |
| Underweight (N,%) | 45 (3.30%) | | -0.012 (-0.059, 0.035) |  | | | -0.005 (-0.015, 0.005) |  | | | | | -0.002 (-0.025, 0.020) |  | | | 0.001 (-0.004, 0.006) |  | | |
| Normal Weight (N,%) | 1030 (58.79%) | | -0.006 (-0.011, -0.001) * | |  | | -0.010 (-0.016, -0.005) * | | | |  | | -0.006 (-0.011, -0.001) * | | |  | -0.005 (-0.010, -0.001) * | |  | |
| Overweight (N,%) | 319 (18.05%) | | -0.017 (-0.024, -0.010) * | |  | | 0.011 (-0.014, 0.036) |  | | | | | -0.001 (-0.012, 0.009) |  | | | -0.006 (-0.015, 0.003) |  | | |
| Obese (N,%) | 374 (19.86%) | | -0.019 (-0.025, -0.012) * | |  | | -0.002 (-0.020, 0.015) |  | | | | | -0.005 (-0.016, 0.006) |  | | | -0.003 (-0.014, 0.008) |  | | |
| Physical activity (hour / week) ^c^ | | | |  | | |  | 0.003 | | | | |  |  | | |  | 0.297 | | |
| Non-activity (N,%) | | 1385(71.92%) | |  | | | -0.017 (-0.023, -0.011) * | | |  | | |  |  | | | -0.012 (-0.018, -0.007) * | | |  |
| 0.1-0.9 (N,%) | 45 (2.99%) | |  |  | | | -0.019 (-0.051, 0.013) |  | | | | |  |  | | | -0.010 (-0.033, 0.013) |  | | |
| 1.0-3.4 (N,%) | 122 (9.44%) | |  |  | | | -0.029 (-0.039, -0.019) * | | |  | | |  |  | | | -0.020 (-0.035, -0.005) * | |  | |
| 3.5-5.9 (N,%) | 58 (4.54%) | |  |  | | | -0.046 (-0.061, -0.031) * | | |  | | |  |  | | | -0.046 (-0.076, -0.017) * | |  | |
| ≥6 (N,%) | 158 (11.12%) | | |  | | | -0.027 (-0.048, -0.007) * | | |  | | |  |  | | | -0.012 (-0.018, -0.005) * | |  | |

^a^ Puberty status was defined as “pubertal” if testosterone ≥ 50 ng/dL in males, estradiol ≥ 20 pg/ml or menstrual period started in females, otherwise puberty status was defined as “prepubertal” ;

^b^ Underweight (BMI < 5th percentile), Normal weight (BMI 5th to < 85th percentiles), Overweight (BMI 85th to < 95th percentiles), Obese (BMI ≥ 95th percentile);

^c^ Physical activity pertains to individuals aged 12 and above; *P* * *P* for interaction; BMI: body mass index; * *P*-value <0.05;

In the subgroup analysis, the 'race', 'education level', and 'blood collection session' subgroups were adjusted for 'age', 'race', 'education level', 'ratio of family income to poverty', 'diabetes', 'blood collection session', and 'cholesterol', excluding the subgroup variable. The same adjustments were made for the 'BMI Categories' and 'physical activity' subgroups.
